# Haglund Deformity – Surgical Resection by the Lateral Approach

**DOI:** 10.5704/MOJ.1503.006

**Published:** 2015-03

**Authors:** S Natarajan, VL Narayanan

**Affiliations:** Department Of Orthopaedics, Saveetha Medical College and Hospital, Thandalam, Kancheepuram district, Tamilnadu, India

**Keywords:** Haglund deformity, Calcaneal Ostectomy, AOFAS Ankle Hind Foot scale

## Abstract

The aim of this study was to analyse the outcome of surgical Haglund deformity is a prominence in the postero superolateral aspect of the calcaneum. Haglund deformity is a prominence in the postero superolateral aspect of the calcaneum, causing a painful bursitis, which may be difficult to treat by non-operative measures alone. Various surgical methods are available for effective treatment of refractory Haglund’s deformity. This study is to evaluate whether adequate resection of Haglund deformity by a lateral approach provides good to excellent results. During the period from 2009 to 2012, 40 patients with 46 feet had undergone resection of Haglund deformity using lateral approach and the outcome was analysed using AOFAS Ankle-Hind Foot Scale. The mean AOFAS score at the follow up was 86/100, with the majority of patients reporting alleviation of pain at one year follow up. The lateral approach to calcaneal ostectomy can be an effective treatment for those suffering from refractory Haglund deformity. However, the patient must be made aware of theduration of recovery being long.

## Introduction

Haglund deformity is described as a prominence of the postero superolateral calcaneum affecting the supero anterior bursa and the Achilles tendon^1^. McGarvey et al reported 89% of their patients improved with non-operative treatment and surgery was indicated for patients not responding to nonoperative treatment^2,3^.

For patients who do not respond to non-operative treatment, there are numerous surgical options like open and endoscopic technique^4,5,6,7,8,9^. The results of surgical treatment have been varied and inconsistent, as to when surgery is indicated and what procedures result in optimal clinical outcomes^10,11,12^. The current study was to determine the outcome of calcaneal tuberosity resection through lateral approach for refractory Haglund deformity.

## Materials and Methods

**Figure 1:**
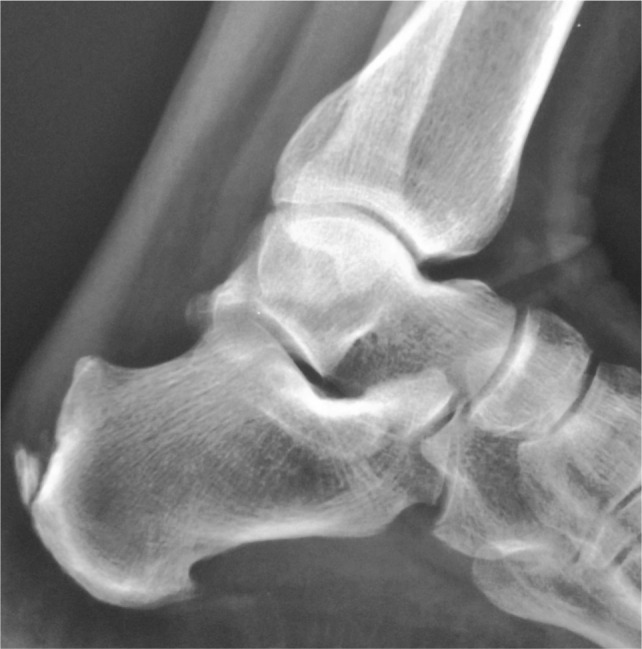
Preoperative radiograph Showing Haglund Deformity.

During 2009 to 2012, we treated 46 cases of Haglund deformity in 40 symptomatic patients at the Saveetha Medical College and Hospital, Chennai by resection of the postero superior calcaneal tuberosity through lateral approach. All 40 patients who were unresponsive to nonoperative treatment for more than 6 months are included in this study. All 46 calcaneal tuberosity resection were performed by a single surgeon and were available for follow up throughout the study. Twenty eight patients were female and twelve patients male. The mean age of the patient was 44 years (range: 38 to 50 years) and the mean follow up time was 13 months (range: 12 to 15 months). Six patients had bilateral operation performed. Preoperative lateral weight bearing radiograph was taken and evaluated for Chauveauxliet angle of more than 12 degrees, parallel pitch lines, presence of retrocalcaneal enthesiophytes^13,14^ and the deformity (Figure 1) for planning the desired angle of ostectomy and the amount of bone to be resected.

Patients who had a lateral approach had a 5 to 8 cm lateral incision along the lateral border of the Achilles tendon insertion. A full-thickness skin flap was made to the tendon. The insertion of the Achilles tendon was identified and resected along the lateral border, exposing the prominent calcar tuber. Using a ½-inch curved osteotome, this was resected and the edges smoothed with a rongeur and rasp.

**Figure 2:**
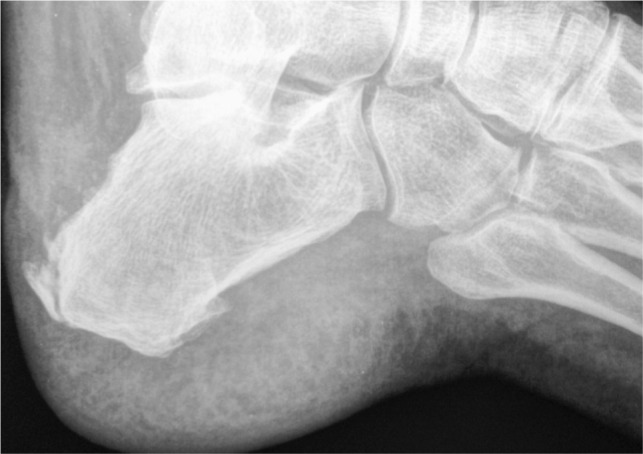
Post Operative radiograph.

In all cases, the dorsal ostectomy was performed initially, allowing exposure of the remaining posterior component, which subsequently was resected and rasped to remove sharp edges. Post-operative radiograph was taken following surgery. (Figure 2)

The AOFAS ankle hind foot scale was employed to evaluate the patient’s outcome. The AOFAS ankle- hind foot score evaluates pain (40 points), function (50 points) and alignment (10 points). It was collected prior to surgery and at the latest post operative follow-up. In addition patients were asked if they would recommend the procedure to others experiencing the same preoperative symptoms.

Patients were evaluated in the hospital at 6 weeks, 3 months, 6 months and 12 months following surgery.

## Results

The mean AOFAS score at the follow-up was 86/100 (range: 60 to 97), an improvement of 28 points from the mean preoperative score. The majority of patients reported alleviation of pain at one year follow-up. Thirty two of the forty patients indicated that they would recommend the procedure to others experiencing the same preoperative symptoms. Of the eight patients that declined to recommend the procedure, four patients reported delayed recovery period, two patients reported mild pain and two patients reported moderate pain at one year follow-up.

The AOFAS score for patients who declined to recommend the procedure was 68 (range: 55 to 97). The delay in recovery period of four patients was 8 to 10 months and the other four patients felt that their pain to be improved from that of the preoperative period and all the four patients described their pain as localized to their heel in the same location at which it had occurred preoperatively.

Three patient had superficial wound infection that promptly responded to antibiotic therapy and dressing.

## Discussion

The treatment of Haglund deformity remains a significant orthopaedic challenge. Many patients may benefit from surgical intervention. The various surgical methods described to treat this deformity have produced mixed results, making it to difficult for physician and patient alike to decide under what circumstances and with what methods to intervene surgically^4,6,15^.

The results of our study suggest that calcaneal ostectomy produces outcome that justify surgical intervention in cases of Haglund deformity not responding for conservative treatment. Mean AOFAS scores for patients in this study were 86/100 and 80% of the patients responded that they would recommend the procedure to others suffering from Haglund deformity.

The results presented are similar to outcomes previously reported by Brunner *et al*^5^ and Sella *et al*^16^ using AOFAS score and Sammarco *et al*^3^ using the Maryland foot score.

The time needed by patients for return to normal activity after surgery for Haglund deformity has been reported. In our study, patients returned to normal function by 6 months following calcaneal ostectomy through lateral approach. Our results are similar to study reported by Saxena *et al*^17^ where the mean return to activity was 15 weeks and Anderson *et al*^18^ reported that patients return to normal activity by 6 months following surgery.

Adequate resection of the bone is required to produce a good clinical outcome. Sella et al highlighted the importance of enough bone being resected to allow decompression of the tendon and the retrocalcaneal bursa^16^.

Adequate resection of the periosteum on the medial side is difficult through lateral approach. Anderson *et al* suggested that tendon splitting approach allows adequate resection of periosteum on the medial side^18^.

## Conclusion

Our study suggests that, lateral approach to calcaneal ostectomy can be an effective treatment for those suffering from refractory Haglund deformity. However the recovery period to obtain a maximum benefit following surgery is longer (6 months). The awareness for longer recovery period should be explained to the patients undergoing calcaneal ostectomy for Haglund deformity.
